# Neoadjuvant sintilimab and chemotherapy for resectable esophageal squamous cell carcinoma: a phase II clinical trial

**DOI:** 10.3389/fimmu.2025.1486275

**Published:** 2025-04-07

**Authors:** Jincheng Guo, Chengrui Qiao, Jiabin Lu, Shiqing Yang, Boyi Zhang, Dongxia Tang, Hui Pang

**Affiliations:** ^1^ Department of Thoracic Surgery, The First Affiliated Hospital of Henan Polytechnic University, Jiaozuo, Henan, China; ^2^ Department of Medical Oncology, The First Affiliated Hospital of Henan Polytechnic University, Jiaozuo, Henan, China

**Keywords:** esophageal squamous cell carcinoma, neoadjuvant therapy, sintilimab, chemotherapy, immunotherapy

## Abstract

**Background:**

Combination of anti-PD-1 monoclonal antibody with chemotherapy has been widely used as a first-line treatment for metastatic esophageal squamous cell carcinoma (ESCC). However, the efficacy of this therapeutic combination as a neoadjuvant intervention for resectable ESCC remains inadequately explored. This study aims to evaluate the efficacy and safety of sintilimab in combination with chemotherapy as a neoadjuvant therapy for ESCC.

**Methods:**

In this single-arm, phase II study, patients with histopathologically diagnosed resectable ESCC who had clinical cT1-3/N0-1M0 (stage II-III) were recruited. Sintilimab (200mg, iv, d1) in combined with chemotherapy (nab-paclitaxel 260 mg/m^2^, d1 and cisplatin 75 mg/m^2^, d1-3) were administered every 3 weeks for 2 cycles. The primary endpoint was pathological complete response (pCR).

**Results:**

From November 2020 through November 2022, 29 patients were enrolled and 27 completed the two cycles of neoadjuvant therapy. A total of 21 patients underwent surgery. The pCR rate was 28.6% (6/21) and the major pathologic response (MPR) rate was 42.9% (9/21). The most common Grade 3 or 4 treatment-related adverse events were leukopenia (26.7%) and neutropenia (20%). No delays in surgical procedures or unexpected surgical complications attributable to the treatment were reported.

**Conclusions:**

The combination of sintilimab and chemotherapy as a neoadjuvant regimen was tolerable and associated with favorable responses for ESCC patients. Given these favorable results, this regimen could serve as a viable alternative in the neoadjuvant treatment landscape for ESCC, with particular applicability to Chinese patient populations.

**Clinical trial registration:**

https://www.chictr.org.cn/, identifier ChiCTR2000040345.

## Introduction

Esophageal cancer (EC) is the seventh most commonly diagnosed cancer and the sixth leading cause of cancer related deaths worldwide (1). The incidence of EC varies geographically, with China being one of the areas with the highest incidence. In 2016, 252,500 new cases of EC were diagnosed, and 193,900 patients died from EC in China (2). Squamous cell carcinoma (SCC) is the main histological subtype in China, making up around 90% of newly diagnosed EC cases.

Esophagectomy is the primary curative treatment for resectable esophageal squamous cell carcinoma (ESCC). Neoadjuvant chemotherapy and chemoradiation are commonly used as standard preoperative treatments to enhance the R0 resection rate and, subsequently, improve long-term survival (3). In OEO2 study, neoadjuvant chemotherapy with cisplatin and fluorouracil moderately increased the R0 resection rate from 54% to 60% and reduced the risk of death and disease progression by 16% and 18%, respectively, compared to surgery alone for patients with ESCC (4, 5). Adding radiotherapy to neoadjuvant chemotherapy appears to further improve treatment effectiveness. The CROSS study demonstrated that preoperative chemoradiation with carboplatin and paclitaxel decreased the risk of death and disease progression by 52% compared with surgery alone. The R0 resection rate achieved 92%, and the pathological complete response was 29% (6).

However, neoadjuvant chemoradiotherapy tends to cause more postoperative complications, which leads to poor tolerance and limits the survival benefit. A randomized clinical trial indicated addition of radiotherapy to neoadjuvant chemotherapy resulted in higher histological complete response rate, higher R0 resection rate, but, had no significantly affect to PFS and OS (7). In fact, the preoperative chemotherapy was more widely used in China due to its better tolerance and lower economic burden. Thus, there is an urgent need for new neoadjuvant treatments with higher pathological response rate, better tolerance and the potential to further improve survival outcomes.

The combination of immune checkpoint inhibitor such as anti-PD-1 monoclonal antibody with platinum-based chemotherapy has become the standard first-line treatment for ESCC. Sintilimab is a novel fully recombinant human IgG4 anti-PD-1 monoclonal antibody. In a randomized phase 3 study (ORIENT-15 study), sintilimab plus chemotherapy showed significant improvements in survival benefit and tumor response in the first line treatment of ESCC compared to placebo. The median progression free survival increased from 5.7 months to 7.2 months (HR = 0.56, p < 0.001). The ORR rose from 45% to 66%. The safety profile of the two treatment regimens were comparable (8). These results demonstrated the potential and feasibility for the regimen of sintilimab plus chemotherapy in ESCC.

This open, single-arm pilot study was performed to explore the efficacy and safety of neoadjuvant sintilimab combined with chemotherapy in resectable ESCC.

## Methods

### Patients

Eligible patients were 18–70 years old and diagnosed with histopathologically confirmed ESCC of clinical stage II/III/IVA. Their condition was considered surgically resectable or potentially resectable by thoracic surgeon. Other key inclusion criteria included an Eastern Cooperative Oncology Group (ECOG) performance status of 0 or 1, adequate organ function, and no history of other malignant tumors. No prior chemotherapy, radiotherapy or immune checkpoint inhibitor were permitted. Patients who existed or had the risk of developing tracheoesophageal fistula or aortoesophageal fistula were excluded.

### Study design

This study was a prospective, single-arm, phase II clinical trial. Eligible patients received intravenous infusions of nab-paclitaxel (260 mg/m^2^, d1), cisplatin (25 mg/m^2^, d1-3) and sintilimab (200mg d1) every 3 weeks for a total of two cycles before surgery. Approximately 4-6 weeks after the final neoadjuvant therapy dose, an esophagectomy (either McKeown esophagogastrectomy or thoracoscopic McKeown esophagogastrectomy) was performed. Postoperative treatment was permitted and determined by the investigator. Dose interruption, delay, or discontinuation of sintilimab due to Grade 3 or 4 AEs was permitted, while dose reduction was not allowed. For chemotherapy, the dose might be reduced in cases of certain high-grade hematologic or non-hematologic toxicities. This study was approved by the Ethics Committee of the First Affiliated Hospital of Henan Polytechnic University (KY2020-11-001). All procedures conducted in this study involving human participants adhered to the Declaration of Helsinki (as revised in 2013) and Good Clinical Practice Guidelines. All enrolled patients gave written informed consent. The trial was registered at chictr.org.cn under the number ChiCTR2000040345.

### Endpoints and assessments

The primary endpoint was pathologic complete response (pCR). The secondary endpoints included major pathological remission (MPR), objective response rate (ORR), and safety. Pathologic response was assessed using the modified Ryan scheme in the College of American Pathologists (CAP) Cancer Protocol for Esophageal Carcinoma. Tumor regression grade (TRG) was classified into four grades: 0 (Complete response): no viable cancer cells, including lymph nodes; 1 (near complete response): single cells or rare small groups of cancer cells; 2 (partial response): residual cancer with evident tumor regression but more than single cells or rare small groups of cancer cells; 3 (poor or no response): extensive residual cancer with no evident tumor regression (1). pCR was defined as TRG 0 and MPR was defined as the sum of pathologic complete response and near pathologic complete response (TRG 0~1). Pathological regression was assessed using hematoxylin and eosin (H&E) stained slides of surgical specimens. Both primary tumor and sampled lymph nodes were evaluated.

ORR was calculated as the sum of patients who achieved tumor complete response (CR) and partial response (PR) according to RECIST 1.1. Tumor response was evaluated based on enhanced CT scan images before and after the completion of neoadjuvant treatment (7 days before surgery). Radiographic surveillance was conducted every 90 days till disease recurrence/progression or death. All pathological data and imaging data were reviewed by two independent pathologists or radiologists. Treatment-related adverse events (TRAEs) and abnormal laboratory findings were reported according to the National Cancer Institute Common Terminology Criteria for Adverse Events, version 5.0.

### Statistical analysis

The sample size was determined using PASS version 15. The pCR of primary esophagus following chemotherapy was approximately 10%. Assuming the neoadjuvant sintilimab plus nab-paclitaxel-cisplatin regimen would achieve a pCR rate of 30%, a sample size of 24 was required to provide 80% power, which was calculated using the one-proportion exact-test with a one-sided type I error of 5%. Considering a dropout rate of 15%, we planned to enroll 29 patients. Pathological response was evaluated in the population who underwent surgery. The safety set included all patients who received at least one dose of the treatment combination. All statistical analyses were performed using SAS 9.4 statistical analysis software.

## Results

Between November 2020 and November 2022, 35 patients were screened and 29 were enrolled. As shown in **Table 1**, 20 patients were male and 9 were female. The median age was 64 years (range, 35-70 years). The most common locations of the primary tumor were the middle esophagus (65.5%), followed by the lower esophagus (27.6%). Only 2 patients (6.9%) had the primary tumor in the upper esophagus. At baseline, 13 patients (44.8%) had stage II disease as defined by the AJCC Eighth Edition, while the others had stage III (55.2%) disease.

**Table 1:** Patient demographic and baseline characteristics.

Characteristics	N = 29
Age, years, median (range)	64 (35-70)
Gender, n (%)	
Male	20 (69.0)
Female	9 (31.0)
Performance score, n (%)	
0	25 (86.2)
1	4 (13.8)
Tumor location, n (%)	
Upper	2 (6.9)
Middle	19 (65.5)
Lower	8 (27.6)
Smoking history, n [%]	20 (69.0)
Drinking history, n [%]	18 (62.1)
Diabetes, n [%]	10 (34.5)
Hypertension, n [%]	8 (27.6)
Tumor	
T1	9
T2	4
T3	16
Node	
N0	8
N1	21
Clinical TNM stage*, n (%)	
II	13 (44.8)
III	16 (55.2)

*Clinical disease stage was assessed according to the criteria of the American Joint Committee on Cancer, Eighth Edition.

27 patients completed the two cycles of neoadjuvant therapy, while two others were lost follow up after the first dose of the regimen. One patient, who developed immune-associated pneumonia and recovered after glucocorticoid treatment, withdrew from the study and continued chemotherapy due to concerns about post-surgery risk. Two patients who achieved a complete radiographic response chose not to receive surgery. Three patients refused surgery in this institution and withdrew their informed consent. In total, 21 patients (72.4%) underwent radical resection.

No treatment-related surgical delays occurred, with a median interval of 32 days (range: 28.5, 41.5) between the last dose of neoadjuvant therapy and surgery. All patients underwent minimally invasive esophagectomy and achieved R0 surgical resection. There were no death or unplanned hospital readmissions within 3 months post-surgery. The operation information is summarized in **Table 2**.

**Table 2:** Surgery information and postoperative complications.

Operation information	
Assessed patients	21
Interval to surgery, median (IQR), days	32.0 (28.5-41.5)
Duration of operation (min)	230.0 (210.0, 432.0)
Intraoperative blood loss (mL)	125.0 ± 34.7
Lymphadenectomy	
Two-field	14 (66.7%)
Three-field	7 (33.1%)
Surgical approach	
MIE	21 (100%)
OE	0
Postoperative complications	
anastomotic leakage	4 (19.0%)
pleural effusion	3 (14.3%)
pneumonia	2 (9.5%)
laryngeal nerve paralysis	2 (9.5%)

For 21 patients who received surgery, 6 (28.6%) reached a pCR. Additionally, 3 (14.3%) had a near pathologic complete response, achieving in a MPR rate of 42.9%. Tumor regression score of the remaining 12 patients was 2. Radiographic evaluation was conducted on 24 patients after 2 treatment cycles. Tumor shrinkage was observed in all patients. According to RECIST v1.1, 4 patients (16.7%) achieved complete response, 13 (54.2%) achieved partial response, and the remaining 7 patients (29.2%) were evaluated as stable disease. Overall, the objective response rate and disease control rate was 70.8% and 100%, respectively.

As shown in **Table 3**, treatment related adverse events (TRAEs) of any grade observed in 25 (86.2%) patients. The most common chemotherapy-related AEs of all grades were alopecia (65.5%), leukopenia (58.6%), and neutropenia (58.6%). The most common immune-related AEs were hypothyroidism (10.3%) and pneumonia (6.9%). Nine patients experienced grade 3 AEs, all of which were identified as treatment-related. Chemotherapy related Grade 3 or 4 AEs included leukopenia (27.6%), neutropenia (20.7%) and thrombocytopenia (3.4%). One patient experienced Grade 3 immune-associated pneumonia after 2 cycles of treatment and recovered with glucocorticoid treatment. No other Grade 3 or 4 immune-related AEs occurred. No patient died in-hospital or within 3 months after surgery, and no severe perioperative complications were reported. Six patients required chemotherapy suspension due to hematological toxicities, and 4 received lower dose of nab-paclitaxel (200 mg/m^2^, d1) and cisplatin (20 mg/m^2^, d1-3) in subsequent cycles. No treatment-related surgical delays occurred.

**Table 3:** Treatment-related adverse events.

TRAE	Incidence, n (%)	
	All grades n=29	Grade 3/4 N=29
All	25 (86.2%)	
Alopecia	19 (65.5)	0
Fatigue	18 (62.1)	0
Leukopenia	17 (58.6)	8 (27.6)
Neutropenia	17 (58.6)	6 (20.7)
Nausea	12 (41.4)	0
Increased alanine transaminase	7 (24.1)	0
Diarrhea	6 (20.7)	0
Vomiting	6 (20.7)	0
Increased aspartate transaminas	6 (20.7)	0
Anemia	5 (17.2)	0
Thrombocytopenia	5 (17.2)	1 (3.4)
Rash	4 (13.8)	0
Hypothyroidism	3 (10.3)	0
Pneumonia	2 (6.9)	1 (3.4)

The postoperative complications are summarized in **Table 3**. The most common complications were anastomotic leakage (4 patients, 19.0%) and pleural effusion (3 patients, 14.3%). All patients recovered with non-surgical intervention. Two patients (9.5%) developed postoperative pneumonia, with one case progressing to sepsis. Both patients recovered after the infusion of broad-spectrum antibiotics. Recurrent laryngeal nerve paralysis was observed in two patients (9.5%). No patient died in-hospital or within 3 months after surgery, and no unexpected surgical complications occurred.

## Discussion

In this prospective, single-arm pilot study, neoadjuvant treatment with sintilimab and chemotherapy (Nab-paclitaxel 260 mg/m^2^, d1 and cisplatin 75 mg/m^2^, d1-3) for 2 cycles in resectable ESCC resulted in a pCR rate of 28.6% and an MPR rate of 42.9%. The radiographic complete response rate according to RECIST 1.1 was 16.7%. There were no surgical delays or mortality within 3 months post-surgery. Eight (38.1%) patients developed surgical complications, all of whom recovered with non-surgical interventions. These results provide preliminarily evidence of the efficacy and safety of our treatment regimen for neoadjuvant treatment of resectable ESCC.

The prognostic significance of pathologic complete response (pCR) after induction therapy in patients with esophageal cancer has been demonstrated in several studies (9, 10). In our study, the pCR and MPR rates were 28.6% and 42.9%, respectively. Since two patients with radiographic complete response and one patient with radiographic partial response did not undergo surgery, and their pathologic response was lost, the actual pCR and MPR rate of our treatment regimen may be higher. As anticipated, the addition of an immune checkpoint inhibitor to chemotherapy significantly increased the pCR rate compared to preoperative chemotherapy alone (11).

The CROSS study (12) promotes concurrent chemoradiotherapy as the standard neoadjuvant therapy for resectable ESCC. Neoadjuvant concurrent chemoradiotherapy has shown a higher pCR rate compared to neoadjuvant chemotherapy and is widely adopted in western countries. However, its accessibility and affordability are limited in same area of China. In our study, the pCR rate was comparable to the CROSS study (28.6% vs. 29%), indicating that neoadjuvant therapy with an immune checkpoint inhibitor and chemotherapy can be a possible alternative. Another advantage of this regimen is its favorable safety profile. Radiotherapy may increase the incidence and severity of certain AEs such as leukopenia, neutropenia and radiation esophagitis. In NEOCRTEC5010 study (13), 109 patients (48.9%) had leukopenia of grade 3 or 4, and 102 (45.7%) had neutropenia of grade 3 or 4. In our study, the incidence of leukopenia of grade 3 or 4 was 27.6% and neutropenia of grade 3 or 4 was and 20.7%. Regarding postoperative complications, our study noted two cases (9.5%) of respiratory complication and 4 cases (19.0%) of anastomotic leakage, all of which recovered with non-surgical intervention. No patients died in hospital or within 90 days post-surgery. Perioperative complication and treatment-related death can undermine the survival benefit of neoadjuvant therapy. A randomized clinical trial involving 181 patients showed no significant PFS and OS benefit when adding radiotherapy to neoadjuvant therapy, despite higher histological complete response and R0 resection rates. This was mainly due to the relatively higher incidence of postoperative complications in neoadjuvant chemoradiotherapy group, especially anastomotic leakage, respiratory issues and increased postoperative mortality (7).

Nab-paclitaxel and cisplatin were identified as a preferred chemotherapy regimen and are widely used in China. Compared with CROSS study (14), high-dose carboplatin (AUC = 2 per weeks) was replaced with a moderated dose of cisplatin in our study. A multicenter, randomized clinical trial involving 321 patients in China compared the efficacy and toxicity of paclitaxel with fluorouracil, cisplatin or carboplatin in definitive chemoradiotherapy for esophageal squamous cell carcinoma (ESCC). The combination of cisplatin with paclitaxel demonstrated the best 3-year OS rate, although it was associated with relatively higher toxicity (15).

In line with other studies (16), the pathologic response was positively correlated with radiographic response. In patients who achieved pCR, two were evaluated as radiographic CR and 4 reached PR. In the remaining 8 patients with radiographic PR, 3 had near pathologic complete response (TGS1). In terms of surgery, all 21 patients successfully underwent McKeown MIE without open surgery, achieving a 100% R0 resection rate. Our average operative time was 230 minutes, and the intraoperative blood loss was 125.0 ± 34.7 mL (mean ± SD), comparable to patients with esophageal cancer who did not receive neoadjuvant treatment. we also observed that after this neoadjuvant treatment, esophageal tumors tended to adhere loosely and were easier to removal from surrounding tissue during surgery. This contrasts with patients who have undergone radiotherapy or neoadjuvant therapy for lung cancer. The NEOSTAR trial (NCT03158129) suggests that due to hilar fibrosis in some patients, separating the blood vessels can be more challenging. This indicates that different cancer types may respond differently to ICIs (17). It also suggests that neoadjuvant sintilimab combined with chemotherapy did not increase the difficulty of the surgery.

This study has some limitations. First, it was an exploratory single-arm study with a small sample size. A randomized controlled study is warranted, especially to compare this regimen with standard neoadjuvant chemoradiotherapy. Second, the follow-up period was short, and the survival data is not mature. In addition, predictive biomarkers will be explored in further studies.

## Conclusions

This phase II clinical study demonstrated promising efficacy and favorable tolerability of neoadjuvant sintilimab and chemotherapy in resectable esophageal squamous cell carcinoma. An encouraging pCR rate and tumor response were observed. These results suggest that the combination of sintilimab and chemotherapy could become an alternative option in the neoadjuvant treatment of resectable esophageal squamous cell carcinoma.

## Data Availability

The raw data supporting the conclusions of this article will be made available by the authors, without undue reservation.
